# *hsp-90* and *unc-45* depletion induce characteristic transcriptional signatures in coexpression cliques of *C. elegans*

**DOI:** 10.1038/s41598-021-91690-6

**Published:** 2021-06-18

**Authors:** Lukas Schmauder, Klaus Richter

**Affiliations:** Center for integrated protein research at the Department of Chemistry, Technische Universität München, Lichtenbergstr. 4, 85748 Garching, Germany

**Keywords:** Functional clustering, Gene regulatory networks, Microarrays, Germline development, RNAi

## Abstract

Nematode development is characterized by progression through several larval stages. Thousands of genes were found in large scale RNAi-experiments to block this development at certain steps, two of which target the molecular chaperone HSP-90 and its cofactor UNC-45. Aiming to define the cause of arrest, we here investigate the status of nematodes after treatment with RNAi against *hsp-90* and *unc-45* by employing an in-depth transcriptional analysis of the arrested larvae. To identify misregulated transcriptional units, we calculate and validate genome-wide coexpression cliques covering the entire nematode genome. We define 307 coexpression cliques and more than half of these can be related to organismal functions by GO-term enrichment, phenotype enrichment or tissue enrichment analysis. Importantly, *hsp-90* and *unc-45* RNAi induce or repress many of these cliques in a coordinated manner, and then several specifically regulated cliques are observed. To map the developmental state of the arrested nematodes we define the expression behaviour of each of the cliques during development from embryo to adult nematode. *hsp-90* RNAi can be seen to arrest development close to the L4 larval stage with further deviations in *daf-16* regulated genes. *unc-45* RNAi instead leads to arrested development at young adult stage prior to the programmatic downregulation of sperm-cell specific genes. In both cases processes can be defined to be misregulated upon depletion of the respective chaperone. With most of the defined gene cliques showing concerted behaviour at some stage of development from embryo to late adult, the “clique map” together with the clique-specific GO-terms, tissue and phenotype assignments will be a valuable tool in understanding concerted responses on the genome-wide level in *Caenorhabditis elegans*.

## Introduction

Nematode-development is a highly complex process that is defined by temporal and spatial events in different tissues and cell types. Therefore simultaneous events are occurring in this process with chronological timing to enable the highly reproducible development program.

HSP-90 (DAF-21) is a molecular chaperone, crucial for the development of vulva, gonads and oocyte maturation as well as ensuring longevity of *C. elegans*^1–3^. It is an indispensable protein, activating and regulating many clients, for example protein kinases, and transcription factors, such as steroid receptors^4–6^. Inhibition of HSP-90, by either RNAi or specific compounds, therefore has the potential to interfere with several pathways. RNAi arrests the nematode development and reduces motility in later larval stages^7,8^. Prominent responses induced after *hsp-90*/*daf-21* RNAi include the heat-shock response, which is known to be suppressed by HSP-90 in most organisms^1,9,10^. Other affected responses are potentially regulated in a more organism-specific manner, like the innate immune response, which is coupled to the heat-shock response in nematodes^11,12^. Interestingly, both of these responses are also dependent on the developmental state of the nematode, with the heat-shock response being barely inducible in early larvae and also in adult aging nematodes^11^. The reason for these correlations is unclear, but it could be supported by assigning genes clearly to individual responses, so that the common principles and regulatory patterns become obvious.

The HSP-90 cofactor UNC-45 participates in the muscle-specific functions of HSP-90. Invertebrates possess a single *unc-45* gene, which is expressed in muscle cells, where UNC-45 performs HSP-90-dependet folding of the myosin motor domain. It further is expressed in non-muscle tissues of early embryos^13–15^. Depletion of the HSP-90-cofactor UNC-45 leads to rather specific morphological changes, like paralysis due to muscle cell defects and sterility in *C. elegans*^7^.

To see, whether these interacting factors influence common pathways, we compare the transcriptional response to depletion of these two proteins by microarray analysis. Microarrays are high-throuhgput analyses yielding a snapshot of the expression status of each represented gene^16^. For *C. elegans* a wealth of data exists, which link different sample conditions to the induction of certain marker genes. Here, as performed before for yeast^17^, we derive and validate genome-wide coexpression cliques and use statistical analyses to define the cliques responding to *hsp-90* and *unc-45* RNAi treatment.

## Material and methods

### Clustering of genes and coexpression clique separation

Construction of the genome-wide coexpression “clique map” for the nematode GPL200 platform was performed as a stepwise procedure as described for the GPL2529 platform of yeast before^17^. In short, all available microarray datasets for the GPL200 platform (Affymetrix C. elegans Genome Array) were obtained from the GEO repository^18^. This included 2243 individual microarray experiments (Supplemental Table 1). These were normalized against each other with the software RMAexpress (Bolstad, 2014; http://rmaexpress.bmbolstad.com/^19^). Based on these normalized values, Pearson’s correlation coefficients were obtained for each probe-probe pair of the 22,620 probes represented on this array type. The resulting list of correlation coefficients was then ranked to generate the ranked coexpression database with information on each probe represented on the GPL200 platform. The probes were then translated from ProbeSetIDs to the given *C. elegans* gene names. Genes, which were represented by more than one ProbeSetID on this array type, which is the case for 8052 ProbeSets, were specifically labelled to allow distinguishing these ProbeSetIDs in later evaluations. Confirming the quality of the ranked lists, in many cases the top coregulated ProbeSets are two ProbeSets reporting on the same gene (data not shown).

The database was then used to generate a network from these ranked lists by connecting the Top11 genes of each list and collecting these connections for all 22,620 genes employing the algorithm accessible on the webserver clusterEX.de^20^. In short, 121 connections were generated from the Top11 genes and added to an extensive list collecting all these pairwise interactions, thereby generating a network. The final network contained more than 600,000 unique gene–gene connections from about 2,700,000 gene–gene correlations. Thus on average a connection was obtained 4.5 times, leading to a network density almost on par with that of our previously generated network for yeast^17^. This genome-wide nematode gene network was then used to extract the individual cliques by isolating high density areas in an automated fashion from the network as described before^17^. Altogether 307 cliques were obtained, with the largest clique containing 1327 genes and the smallest clique containing 5 genes. The nematode analysis methods are added to the webserver functionality.

### GO-term, phenotype and tissue enrichment

GO-term enrichment was analysed to test, whether some of the 307 aforementioned cliques enrich genes with functional similarity. To this end also published information from phenotype and tissue enrichment studies was used. As such the associations between genes and GO-terms were obtained from the “go_dictionary.csv” table available from https://github.com/dangeles/TissueEnrichmentAnalysis^21^. For phenotype enrichment the table “phenotype_ontology.csv” was employed (PEA^22^) and for tissue enrichment the tissue sets designated as “genesets_golden” were utilized. In all cases the calculation of the enrichment was performed as described^17^ (Supplemental Table 2). 20 randomly scrambled clique sets were generated to determine, whether enrichment is considered relevant up to p-values of 1e-3, 1e-4 or beyond 1e-5.

### Gene-expression changes after RNAi against *hsp-90* and *unc-45*

RNAi was used to deplete nematodes of *hsp-90* or *unc-45* mRNA and to induce the growth arrest and the transcriptomic responses of the nematodes. RNAi-treated nematodes were washed off the plates and were shock frozen immediately. Microarray experiments were performed at the Zentrum für Fluoreszente Proteinanalytik in Regensburg. To study the response to *hsp-90* RNAi or *unc-45* RNAi we analysed independent biological replicates. In these experiments, RNAi did not always yield the same level of growth arrest in the case of *hsp-90*, where the first microarray experiment produced a weaker response. We used each experiment sample/control-pair to assign all its differential expression values to the coexpression cliques and analysed those in respect to significant induction or repression. As the RNAi experiments were analysed on the more rarely employed GPL19230 Affymetrix platform (Affymetrix C. elegans Gene 1.0 ST Array), we bridged the cliques obtained from GPL200 ProbeSets to the GPL19230 ProbeSets. This bridging was performed by employing the given gene names without the ProbeSet-specific indexing. If a gene was represented by more than one ProbeSet in the cliques, then each of those instances was given the value determined from the GPL19230 expression data. If on the other hand, only one GPL200-derived ProbeSet was present in the cliques and several ProbeSets for this gene are recoded on the GPL19230 arrays, then the GPL19230-values were averaged and this value was used to color the clique map and to derive the statistical parameters for the clique. If the same gene contained two different probes on both platforms, then the averaged GPL19230-value was used in both occurences in the clique map. 1603 ProbeSets of the “clique map” did not receive data from GPL19230 this way and had to be omitted in the analysis. Despite these bridging needs between the platforms, significant changes in many cliques can be detected in each analysed RNAi experiment. The observed experiments were also analysed with the Transcriptome Analysis Console (TAC, Thermo Fisher Scientific) as a state-of-art method for analysis of microarray data.

Statistical analysis employing the clique map was done as described before^17^. In short, color coding of the clique set figures was done by determining the differential values for each gene and then assigning discrete values between  − 4 and + 4 for the transcriptional changes of log2 <  − 1 to log2 >  + 1. For each discrete value a red tone or blue tone was defined in Cytoscape (https://cytoscape.org/)^23^. In cases where responses were weak, like both *unc-45* experiments, the scale was adjusted to reach from log2 <  − 0.25 to log2 >  + 0.25. This analysis leads to information on most cliques as to whether they are induced or repressed with statistical significance as described before^17^. This method to evaluate nematode array data will be implemented for public use in the clusterEX.de webserver, which currently has this functionality only for yeast arrays.

Correlation analysis between different samples was made by plotting the cliques’ expression values against each other and obtaining the coefficient of determination R^2^ for the regression line. If R^2^ was closer to 1, the correlation between the two sets was considered to be better. These results were compared to correlations on the gene level in cases where identical array types were utilized.

### Analysis of microarray data on development

To define moments of clique relevance during development, time points from developmental series were used to determine a transcriptional status for each clique in the map. In many cases, cliques react to developmental steps as concerted units resulting in a non-random distribution of up- and downregulated hits throughout the 307 cliques. To cover several larval states, three published GPL200 series were obtained from the GEO repository. These represent a time course of early development with data from embryo, L1 and L4 larvae (GSE6547^24^) and a time course describing the aging process with time points at L4 larvae, and adults at day6 and day15 of development (GSE21784^25^). Lastly, a time-course was included describing different stages of larval development, composed of L3, L3-lethargus, L4, L4-lethargus and young adult (GSE46291^26^). Expression values were obtained from the normalized data table containing all public GPL200 experiments (see above).

## Results

### A genome-wide coexpression clique map for the nematode *C. elegans*

To obtain transcriptional units influenced by *hsp-90* and *unc-45* RNAi-treated nematodes, we first generated gene cliques that are coregulated in *C. elegans*, in which each of the 22,620 genes is assigned to exactly one clique. We had used the same procedure before to generate a coexpression clique map for *S. cerevisiae*^17^. Based on the same stepwise procedure, we grouped every gene reported on standard microarrays of the GPL200 platform into one coexpression clique of at least five genes. The procedure resulted in 307 coexpression cliques, which were visualized in Cytoscape to generate the “coexpression clique map” for *C. elegans*. We set out to test, whether these 307 coexpression cliques are gene groups with a high level of functional similarity, as it was observed for the yeast clique map before^17^. Therefore, we investigated all cliques by GO-term enrichment analysis. 220 of the 307 cliques show a GO-term enrichment with a p-value lower than 1e–3, 172 showed less than 1e–4, and 148 of the 307 cliques showed p-values of less than 1e–5 (Best results in Table 1). This is far better than 307 random cliques, which yielded these p-values 18 times, 2 times and zero times. We also found significant enrichments employing phenotype enrichment analysis (PEA^22^) and tissue enrichment analysis (TEA^21^) with 145, 106 and 81 cliques being enriched for the same phenotype (20, 3 and 0 times in random cliques) and 45, 37 or 29 cliques being enriched for tissue-specific expression in the three p-value categories (0, 0, and 0 times in random cliques). The values also are far better than cliques composed of random genes. In this way, roughly two thirds of the 307 coexpression cliques were assigned either a function, a related phenotype or a tissue-specific expression with acceptable significance criteria of below 1e–4 (Table 1).

**Table 1:** Most relevant coexpression cliques of the clique map, their size and position, GO-term assignment, phenotype enrichment and tissue enrichment.

Clusternumber	Cluster name	Clique position	Best GO-term	log10(pvalue)_GO	Enrichment-Fold_GO	Best PEA-Term	log10(pvalue)_PEA	Enrichment-Fold_PEA	Best TEA-Term	log10(pvalue)_TEA	Enrichment-Fold_TEA
80	rps-14_21270-rps-11_20714	R4 C17	Structural constituent of ribosome GO:0003735	189.66	83.29	Pleiotropic defects severe early emb WBPhenotype:0,000,270	120.69	43.09	WBPaper00026980_intestine_enriched_WBbt_0005772_1970	1.44	1.28
211	srj-42-srw-113	R1 C1	Sensory perception GO:0007600	143.77	6.31	Dauer metabolism variant WBPhenotype:0,001,547	10.86	2.84	WBPaper00040420_FLP_enriched_WBbt_0006828_288	0.00	0.46
282	srj-21-srh-32	R1 C3	Intrinsic component of membrane GO:0031224	128.75	2.29	Dauer metabolism variant WBPhenotype:0,001,547	7.14	3.04	WBPaper00040420_FLP_enriched_WBbt_0006828_288	0.00	0.37
187	F45H10.2-R53.4_21676	R3 C4	Organelle Inner membrane GO:0019866	74.24	34.23	Avoids bacterial lawn WBPhenotype:0,000,402	27.00	6.57	WBPaper00026980_intestine_enriched_WBbt_0005772_1970	3.37	1.50
93	flp-5-F17C11.2	R4 C4	Neuropeptide signaling pathway GO:0007218	56.65	67.09	Sinusoidal movement variant WBPhenotype:0,004,018	22.79	11.85	WBPaper00037950_all-neurons_larva_enriched_WBbt_0003679_1013	22.28	3.93
90	ckr-1-T09B9.3	R1 C5	Intrinsic component of membrane GO:0031224	52.94	1.97	Sinusoidal movement variant WBPhenotype:0,004,018	8.60	3.85	WBPaper00037950_all-neurons_larva_enriched_WBbt_0003679_1013	53.78	4.32
42	rab-28-jbts-14_9542	R3 C5	Cell projection assembly GO:0030031	45.54	56.43	Amphid phasmid sensillum morphology variant WBPhenotype:0,001,527	16.40	17.48	WBPaper00037950_BAG-neuron_embryo_enriched_WBbt_0006825_454	42.10	7.48
203	sre-33-ZK1025.1_8337	R1 C2	Sensory perception GO:0007600	45.42	4.04	Dauer metabolism variant WBPhenotype:0,001,547	6.13	2.24	WBPaper00037950_coelomocytes_larva_enriched_WBbt_0005751_229	0.02	0.64
84	col-84-col-45	R9 C17	Collagen trimer GO:0005581	44.38	77.74	Dumpy WBPhenotype:0,000,583	3.04	8.11	WBPaper00040420_FLP_enriched_WBbt_0006828_288	0.00	0.20
41	col-117-col-167_1015	R9 C25	Structural constituent of cuticle GO:0042302	39.93	83.10	Dumpy WBPhenotype:0,000,583	5.47	7.68	WBPaper00037950_germline-precursors_embryo_enriched_WBbt_0006849_974	0.00	0.33
233	his-46_959-his-64	R10 C26	DNA packaging complex GO:0044815	39.57	191.32	Sister chromatid segregation defective early emb WBPhenotype:0,000,772	26.48	94.19	WBPaper00037950_germline-precursors_embryo_enriched_WBbt_0006849_974	0.00	0.07
215	col-138-col-49	R9 C23	Structural constituent of cuticle GO:0042302	35.30	87.06	Blistered WBPhenotype:0,000,025	5.77	41.59	WBPaper00037950_coelomocytes_larva_enriched_WBbt_0005751_229	0.07	0.60
80	rps-14_21270-rps-11_20714	R4 C17	Structural constituent of ribosome GO:0003735	189.66	83.29	Pleiotropic defects severe early emb WBPhenotype:0,000,270	120.69	43.09	WBPaper00026980_intestine_enriched_WBbt_0005772_1970	1.44	1.28
31	pbs-3_18439-rpn-5	R3 C7	Modification-dependent macromolecule catabolic process GO:0043632	23.08	13.30	Meiosis defective early emb WBPhenotype:0,001,041	32.03	23.37	WBPaper00031003_24hr_muscle_enriched_WBbt_0003675_918	2.78	1.65
187	F45H10.2-R53.4_21676	R3 C4	Organelle inner membrane GO:0019866	74.24	34.23	Avoids bacterial lawn WBPhenotype:0,000,402	27.00	6.57	WBPaper00026980_intestine_enriched_WBbt_0005772_1970	3.37	1.50
233	his-46_959-his-64	R10 C26	DNA packaging complex GO:0044815	39.57	191.32	Sister chromatid segregation defective early emb WBPhenotype:0,000,772	26.48	94.19	WBPaper00037950_germline-precursors_embryo_enriched_WBbt_0006849_974	0.00	0.07
93	flp-5-F17C11.2	R4 C4	Neuropeptide signaling pathway GO:0007218	56.65	67.09	Sinusoidal movement variant WBPhenotype:0,004,018	22.79	11.85	WBPaper00037950_all-neurons_larva_enriched_WBbt_0003679_1013	22.28	3.93
2	rps-5_2365-rpl-15_1655	R10 C23	Structural constituent of ribosome GO:0003735	24.02	68.88	Pleiotropic defects severe early emb WBPhenotype:0,000,270	19.08	40.82	WBPaper00037950_PVD-OLL-neurons_larva_enriched_WBbt_0006831_878	0.00	0.23
143	his-20_965-his-4	R11 C22	DNA packaging complex GO:0044815	30.10	191.32	Sister chromatid segregation defective early emb WBPhenotype:0,000,772	18.37	87.98	WBPaper00024505_pharyngeal_enriched_WBbt_0003681_329	0.00	0.18
96	mup-2-unc-87_284	R3 C13	Myofibril GO:0030016	31.32	27.72	Muscle system morphology variant WBPhenotype:0,000,603	17.34	8.57	WBPaper00031003_0hr_muscle_enriched_WBbt_0003675_761	20.71	3.64
218	H28G03.5_1027-H28G03.5_1042	R11 C24	Cell recognition GO:0008037	18.80	92.91	Axon fasciculation variant WBPhenotype:0,000,632	17.15	63.29	WBPaper00045521_Spermatogenic_WBbt_0005784_2743	0.00	0.33
42	rab-28-jbts-14_9542	R3 C5	Cell projection assembly GO:0030031	45.54	56.43	Amphid phasmid sensillum morphology variant WBPhenotype:0,001,527	16.40	17.48	WBPaper00037950_BAG-neuron_embryo_enriched_WBbt_0006825_454	42.10	7.48
234	pcn-1-cyb-3_17196	R7 C12	DNA replication GO:0006260	13.16	29.41	Cytokinesis variant WBPhenotype:0,002,408	16.17	11.19	WBPaper00037950_pharyngeal-muscle_embryo_enriched_WBbt_0005451_598	0.01	0.48
197	unc-11_429-unc-11_430	R12 C11	Phosphatidylinositol binding GO:0035091	15.76	172.19	Mid larval lethal WBPhenotype:0,000,116	15.12	160.50	WBPaper00037950_coelomocytes_embryo_enriched_WBbt_0005751_570	0.09	0.76
1	Y40H4A.2-ZK1053.2	R1 C6	Phosphorus metabolic process GO:0006793	17.12	3.03	Spermatogenesis variant WBPhenotype:0,000,670	1.48	4.25	WBPaper00045521_Spermatogenic_WBbt_0005784_2743	165.27	4.57
20	T22D1.5-inx-14	R2 C1	Embryo development GO:0009790	13.09	1.97	Aneuploidy WBPhenotype:0,001,882	11.50	6.39	WBPaper00037950_germline-precursors_embryo_enriched_WBbt_0006849_974	73.49	5.82
273	C01G10.14-dct-9_3227	R2 C3	Regulation of cell shape GO:0008360	20.29	18.38	Spermatogenesis variant WBPhenotype:0,000,670	2.62	7.10	WBPaper00045521_Spermatogenic_WBbt_0005784_2743	71.95	3.62
111	mlt-9_22518-F33D4.6_14044	R2 C6	Cuticle development GO:0042335	16.79	11.64	Molt variant WBPhenotype:0,002,041	12.53	5.66	WBPaper00037950_hypodermis_embryo_enriched_WBbt_0005733_734	56.15	6.08
90	ckr-1-T09B9.3	R1 C5	Intrinsic component of membrane GO:0031224	52.94	1.97	Sinusoidal movement variant WBPhenotype:0,004,018	8.60	3.85	WBPaper00037950_all-neurons_larva_enriched_WBbt_0003679_1013	53.78	4.32
42	rab-28-jbts-14_9542	R3 C5	Cell projection assembly GO:0030031	45.54	56.43	Amphid phasmid sensillum morphology variant WBPhenotype:0,001,527	16.40	17.48	WBPaper00037950_BAG-neuron_embryo_enriched_WBbt_0006825_454	42.10	7.48
213	xbx-3-DH11.5_20397	R1 C12	Signaling GO:0023052	15.19	2.38	Backward point velocity increased WBPhenotype:0,002,325	7.12	8.29	WBPaper00037950_all-neurons_larva_enriched_WBbt_0003679_1013	41.49	4.53
155	F42A9.7-T22B3.3	R3 C11	Regulation of cell shape GO:0008360	9.67	17.73	Dauer metabolism variant WBPhenotype:0,001,547	1.51	2.28	WBPaper00045521_Spermatogenic_WBbt_0005784_2743	25.38	2.62
83	sol-1-jnk-1_18695	R1 C9	Nervous system development GO:0007399	21.52	4.87	Synapse morphology variant WBPhenotype:0,000,616	10.59	8.04	WBPaper00031532_Larva_Pan_Neuronal_Enriched_WBbt_0003679_1603	25.10	2.45
93	flp-5-F17C11.2	R4 C4	Neuropeptide signaling pathway GO:0007218	56.65	67.09	Sinusoidal movement variant WBPhenotype:0,004,018	22.79	11.85	WBPaper00037950_all-neurons_larva_enriched_WBbt_0003679_1013	22.28	3.93
96	mup-2-unc-87_284	R3 C13	Myofibril GO:0030016	31.32	27.72	Muscle system morphology variant WBPhenotype:0,000,603	17.34	8.57	WBPaper00031003_0hr_muscle_enriched_WBbt_0003675_761	20.71	3.64
34	dhs-28_22199-acs-14	R4 C9	Oxoacid metabolic process GO:0043436	14.25	9.18	Lipid metabolism variant WBPhenotype:0,000,725	4.43	3.34	WBPaper00037950_intestine_embryo_enriched_WBbt_0005772_886	20.41	3.78

### *hsp-90* RNAi affects embryo development and induces stress responses

**Figure 1:**
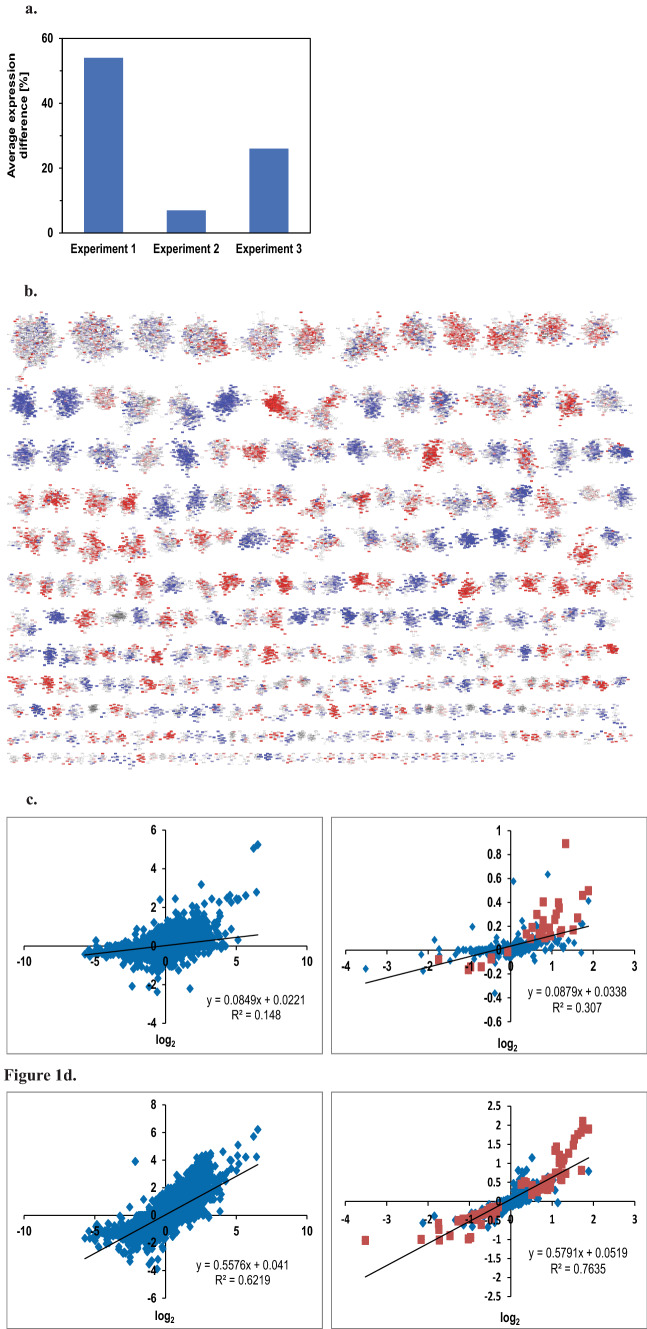
*hsp-90* RNAi affects embryo development and induces stress responses. (**a**) Average gene expression difference of the experiments, determined with TAC, compared to the control RNAi. (**b**) Clique map for experiment 1 of *hsp-90* RNAi versus young adult of control RNAi. A more detailed description of the most strongly affected cliques can be found in Table 2 and corresponds to the positions in the clique map. (**c**) Comparison between experiment 1 and 3 on a gene-by-gene basis (left panel) and comparison between experiment 1 and 3 on a clique-by-clique basis (right panel). (**d**) Comparison between experiment 1 and 2 on a gene-by-gene basis (left panel) and comparison between experiment 1 and 2 on a clique-by-clique basis (right panel). The clique maps of experiment 3 and 2 are shown as Supplemental Fig. 1b and c. Cliques colored in red are induced upon RNAi while cliques in blue are repressed. The linear regression function was generated with Microsoft Excel without weighting, a square value of 1 would indicate a perfect correlation between the cliques.

Having confirmed that the “clique map” of coexpressed genes also holds information on functional, phenotypic and tissue-specific signatures, we set out to investigating the transcriptional response of *hsp-90* RNAi-treated nematodes. We previously had analysed these microarray data based on the Top250 differential regulated genes obtained from three experiments^11^. *hsp-90* depleted nematodes showed sterility, incomplete development of gonad arms and the formation of endomitotic oocytes^7,27^. Development is mostly blocked at a later larval stage. TAC analysis revealed many genes with substantially different expression levels and showed the strongest response in the experiment 1 (P152), while the experiment 2 (A966) and 3 (P062) showed a weaker response (Fig. 1a). Gene expression changes had implied the induction of the heat-shock response and the innate immune response in analyses before^11^, but due to the focus on only 500 of the 22,620 genes measured with this array type, information from the many weaker affected genes could not be considered in this study^11^.

The genome-wide gene expression cliques as defined here, instead allow visualization and analysis of all values. Significance analysis showed enrichment of up- and downregulation in many cliques for experiment 1 (Fig. 1b), but also for the other experiments 2 and 3 (Supplemental Fig. 1b and c). Indeed almost half of the cliques respond to the RNAi-treatment with a concerted response of their genes (Best cliques in Table 2). We first determined the upregulated cliques: these contain the clique col-138-col-49, which is holding genes related to the “structural constituent of cuticle” and the clique abu-7-abu-8_22491 related to the “response to unfolded protein”. The largest upregulated clique, containing 209 genes, is mlt-9_22518-F33D4.6_14044 (“cuticle development” with phenotype of “molt variant” and localized in the “embryo hypodermis”) and other cliques related to cuticle formation, including R12E2.14_75-R12E2.15, col-117-col-167_1015, col-146-col-133, col-128-cdh-10_9234 (enriched in “peptidase activity”) and sqt-2-dpy-9. Cliques which are strongly upregulated also in experiment 2, include the cliques related to the “immune system response” C10C5.2-Y58A7A.3 and K08D10.9-F46A8.1. Based on the assignment of GO-terms, phenotypes and tissues, the largest and strongest downregulated cliques (Table 2) represent “embryo development” (T22D1.5-inx-14, enriched phenotype of “aneuploidy” and localized in “embryonic germline precursors”), “embryo development” (T24D1.3-egg-1, enriched phenotype of “polar body defective early embryo”) and “reproduction” (puf-3-oma-2_18268, enriched phenotype “meiotic chromosome segregation variant” localized in the “germline_precursors”) among many other cliques hinting at the stalled gonad development in agreement with the sterility phenotype observed. Comparing the three experiments a weak correlation can be found between experiment 2 and 1 (R^2^ = 0.148) on a gene-by-gene level, which is increased, if cliques are compared (R^2^ = 0.307, Fig. 1c). The same trend can be seen between experiments 3 and 1 correlating with R^2^ = 0.622 for a gene-to-gene comparison, which increases to R^2^ = 0.764, if cliques are compared (Fig. 1d). Moreover the significance analysis employing 20 random cliques shows that the most strongly up- and downregulated cliques also are usually fulfilling the 1e-5 significance criterium in the compared experiments (Fig. 1c and d, colored in red).

**Table 2:** Most strongly affected cliques by *hsp-90* RNAi and their characteristics.

Cluster name	Clique position	Best GO-Term	Best PEA-Term	Best TEA-Term	Mean STD	Log10(p) Exp 1	Log10(p) Exp 2	Log10(p) Exp 3
col-138-col-49	R9 C23	Structural constituent of cuticle GO:0042302	Blistered WBPhenotype:0000025	WBPaper00037950_coelomocytes_larva_enriched_WBbt_0005751_229	1.43 ± 0.71	28.5	35.6	70.2
abu-7-abu-8_22491	R8 C26	Response to unfolded protein GO:0006986	Dauer constitutive WBPhenotype:0000012	WBPaper00024505_pharyngeal_enriched_WBbt_0003681_329	1.43 ± 0.66	38.9	38.4	51.4
agmo-1_5527-F53B1.4	R10 C14	Pyridoxal phosphate binding GO:0030170	Molt variant WBPhenotype:0002041	WBPaper00037950_hypodermis_embryo_enriched_WBbt_0005733_734	1.29 ± 0.76	14.4	3.2	48.0
bus-8_3160-K04H4.2_2324	R9 C2	Amino sugar metabolic process GO:0006040	Molt variant WBPhenotype:0002041	WBPaper00024505_pharyngeal_enriched_WBbt_0003681_329	1.25 ± 0.73	30.4	3.5	103.9
R12E2.14_75-R12E2.15	R6 C9	Structural constituent of cuticle GO:0042302	Dumpy WBPhenotype:0,000,583	WBPaper00037950_germline-precursors_embryo_enriched_WBbt_0006849_974	1.22 ± 0.67	51.2	21.3	182.3
mlt-9_22518-F33D4.6_14044	R2 C6	Cuticle development GO:0042335	Molt variant WBPhenotype:0002041	WBPaper00037950_hypodermis_embryo_enriched_WBbt_0005733_734	1.08 ± 0.72	137.7	2.6	133.0
R12A1.3-M195.2	R6 C19	Amino sugar metabolic process GO:0006040	Dauer constitutive WBPhenotype:0000012	WBPaper00024505_pharyngeal_enriched_WBbt_0003681_329	1.05 ± 0.63	42.2	14.3	46.7
hsp-16.2-F44E5.4_19238	R12 C4	Response to heat GO:0009408	cadmium response variant WBPhenotype:0001653	WBPaper00037950_coelomocytes_larva_enriched_WBbt_0005751_229	1.03 ± 0.62	3.6	6.2	1.7
lys-3-tsp-1	R11 C40	Carbohydrate metabolic process GO:0005975	Male nervous system development variant WBPhenotype:0001008	WBPaper00040420_ALM_PLM_enriched_WBbt_0005406_198	0.99 ± 0.25	6.4	28.8	6.0
col-117-col-167_1015	R9 C25	Structural constituent of cuticle GO:0042302	Dumpy WBPhenotype:0000583	WBPaper00037950_germline-precursors_embryo_enriched_WBbt_0006849_974	0.94 ± 0.47	10.6	10.3	20.0
C38C6.3-acdh-6	R6 C14	Intrinsic component of membrane GO:0031224	Intestinal vacuole WBPhenotype:0001428	WBPaper00037950_hypodermis_embryo_enriched_WBbt_0005733_734	0.91 ± 0.59	71.0	1.4	59.3
pqn-54-abu-9	R6 C11	Response to unfolded protein GO:0006986	Shortened life span WBPhenotype:0001171	WBPaper00024505_pharyngeal_enriched_WBbt_0003681_329	0.91 ± 0.4	37.6	43.3	33.7
col-146-col-133	R9 C28	Structural constituent of cuticle GO:0042302	Dumpy WBPhenotype:0000583	WBPaper00037950_coelomocytes_larva_enriched_WBbt_0005751_229	0.89 ± 0.46	10.0	6.5	26.7
C36C5.12-F57G8.7	R11 C9	Negative regulation of proteolysis GO:0045861	Male tail morphology variant WBPhenotype:0000070	WBPaper00037950_coelomocytes_larva_enriched_WBbt_0005751_229	0.83 ± 0.71	13.9	1.3	5.0
col-128-cdh-10_9234	R3 C12	Peptidase activity GO:0008233	Molt variant WBPhenotype:0002041	WBPaper00037950_hypodermis_larva_enriched_WBbt_0005733_1250	0.82 ± 0.56	66.1	0.6	61.0
ptr-23_236-ptr-23_16340	R12 C6	Male sex differentiation GO:0046661	Developmental pigmentation variant WBPhenotype:0001009	WBPaper00037950_hypodermis_embryo_enriched_WBbt_0005733_734	0.79 ± 0.56	6.0	0.2	9.5
C10C5.2-Y58A7A.3	R4 C11	Immune system process GO:0002376	Cadmium response variant WBPhenotype:0001653	WBPaper00037950_coelomocytes_larva_enriched_WBbt_0005751_229	0.77 ± 0.31	36.6	83.0	39.9
sqt-2-dpy-9	R9 C6	Structural constituent of cuticle GO:0042302	Dumpy WBPhenotype:0000583	WBPaper00037950_hypodermis_larva_enriched_WBbt_0005733_1250	0.76 ± 0.48	13.0	1.0	18.9
dos-2-grd-2	R8 C7	Extracellular region GO:0005576	Pericellular component morphology variant WBPhenotype:0000912	WBPaper00037950_hypodermis_embryo_enriched_WBbt_0005733_734	0.75 ± 0.52	15.4	0.3	36.4
K08D10.9-F46A8.1	R11 C42	Immune system process GO:0002376	Actin organization biogenesis variant WBPhenotype:0001587	WBPaper00037950_excretory-cell_larva_enriched_WBbt_0005812_528	0.72 ± 0.12	3.7	12.9	2.2
vit-2-vit-4_22519	R12 C22	Extracellular region GO:0005576	Pathogen susceptibility increased WBPhenotype:0001013	WBPaper00037950_pharyngeal-muscle_embryo_enriched_WBbt_0005451_598	− 1.57 ± 1.42	47.9	2.5	5.7
C17E7.4-T06D4.1	R5 C13	ribonucleoprotein granule GO:0035770	P granule defective WBPhenotype:0001301	WBPaper00037950_pharyngeal-muscle_embryo_enriched_WBbt_0005451_598	− 1.07 ± 0.87	168.7	1.2	72.1
171971_x_at-D1054.11_184	R11 C37	Cell GO:0005623	Egg laying defective WBPhenotype:0000006	WBPaper00037950_hypodermis_larva_enriched_WBbt_0005733_1250	− 0.96 ± 0.84	1.4	0.7	0.6
sea-1-R04D3.4	R7 C13	Nucleoside-triphosphatase regulator activity GO:0060589	Embryonic development variant WBPhenotype:0000749	WBPaper00037950_hypodermis_embryo_enriched_WBbt_0005733_734	− 0.93 ± 0.69	39.7	4.0	53.7
T24D1.3-egg-1	R7 C17	Embryo development GO:0009790	Polar body defective early emb WBPhenotype:0001147	WBPaper00037950_GABAergic-motor-neurons_larva_enriched_WBbt_0005190_132	− 0.82 ± 0.72	36.0	0.3	24.0
C46C2.5_15926-W03F11.1	R9 C22	Carbohydrate binding GO:0030246	Apoptosis increased WBPhenotype:0000183	WBPaper00037950_GABAergic-motor-neurons_embryo_enriched_WBbt_0005190_361	− 0.82 ± 0.57	15.7	2.5	13.8
ZC373.2-Y62H9A.6_1596	R3 C18	Flavonoid metabolic process GO:0009812	Cell membrane organization biogenesis variant WBPhenotype:0001982	WBPaper00037950_dopaminergic-neurons_larva_enriched_WBbt_0006746_1230	− 0.8 ± 0.7	53.5	6.0	32.5
ZK1053.4-C08F1.6	R4 C16	Embryo development GO:0009790	Embryonic development variant WBPhenotype:0000749	WBPaper00037950_hypodermis_embryo_enriched_WBbt_0005733_734	− 0.73 ± 0.4	70.9	12.9	40.5
T05E12.6_12439-T05E12.6_12396	R10 C3	Lipid catabolic process GO:0016042	Transgene expression increased WBPhenotype:0001236	WBPaper00037950_pharyngeal-muscle_embryo_enriched_WBbt_0005451_598	− 0.72 ± 0.82	28.7	2.8	3.3
fbxc-28-sdz-28	R7 C8	Modification-dependent macromolecule catabolic process GO:0043632	L1 larval development variant WBPhenotype:0000751	WBPaper00037950_hypodermis_embryo_enriched_WBbt_0005733_734	− 0.69 ± 0.39	26.3	23.5	41.6
K09D9.12-T10C6.10	R10 C7	Protein polyubiquitination GO:0000209	Fat content reduced WBPhenotype:0001183	WBPaper00037950_germline-precursors_embryo_enriched_WBbt_0006849_974	− 0.62 ± 0.54	7.6	1.2	3.8
puf-3-oma-2_18268	R5 C14	Reproduction GO:0000003	Meiotic chromosome segregation variant WBPhenotype:0001499	WBPaper00037950_germline-precursors_embryo_enriched_WBbt_0006849_974	− 0.6 ± 0.53	24.0	0.8	12.3
172276_x_at-Y116F11B.10_466	R12 C20	Chromosome segregation GO:0007059	Rachis wide WBPhenotype:0001943	WBPaper00036375_enriched_in_PVD_OLL_WBbt_0006831_2180	− 0.59 ± 0.47	1.2	0.2	1.0
C41G7.3_2766-ani-2_2946	R8 C2	Multi-organism reproductive process GO:0044703	Cytokinesis variant WBPhenotype:0002408	WBPaper00037950_germline-precursors_embryo_enriched_WBbt_0006849_974	− 0.59 ± 0.51	26.0	0.8	9.0
T22D1.5-inx-14	R2 C1	Embryo development GO:0009790	Aneuploidy WBPhenotype:0001882	WBPaper00037950_germline-precursors_embryo_enriched_WBbt_0006849_974	− 0.54 ± 0.48	122.7	2.0	60.1
let-99_22121-B0238.9_11154	R6 C21	Organelle fission GO:0048285	Embryonic development variant WBPhenotype:0000749	WBPaper00037950_germline-precursors_embryo_enriched_WBbt_0006849_974	− 0.52 ± 0.44	16.6	0.2	15.4
Y116A8C.19-F38C2.7	R10 C25	Poly(A) RNA binding GO:0044822	Dauer metabolism variant WBPhenotype:0001547	WBPaper00040420_FLP_enriched_WBbt_0006828_288	− 0.49 ± 0.25	4.8	5.5	4.5
daf-18_2911-ced-2_4092	R8 C23	Nuclear transport GO:0051169	Cell death variant WBPhenotype:0000729	WBPaper00037950_GABAergic-motor-neurons_larva_enriched_WBbt_0005190_132	− 0.48 ± 0.4	6.9	0.6	10.0
pcn-1-cyb-3_17196	R7 C12	DNA replication GO:0006260	Cytokinesis variant WBPhenotype:0002408	WBPaper00037950_pharyngeal-muscle_embryo_enriched_WBbt_0005451_598	− 0.47 ± 0.38	17.2	1.5	5.3
C10C5.3-C10C5.5	R12 C40	Oxoacid metabolic process GO:0043436	Dauer constitutive WBPhenotype:0000012	WBPaper00037950_pharyngeal-muscle_embryo_enriched_WBbt_0005451_598	− 0.47 ± 0.48	2.6	5.1	2.2

Clique positions (letter = row, number = position from left to right) correspond to the clique map shown in Fig. 1b.

### *unc-45* RNAi leads to delayed conclusion of sperm and vulva development

**Figure 2:**
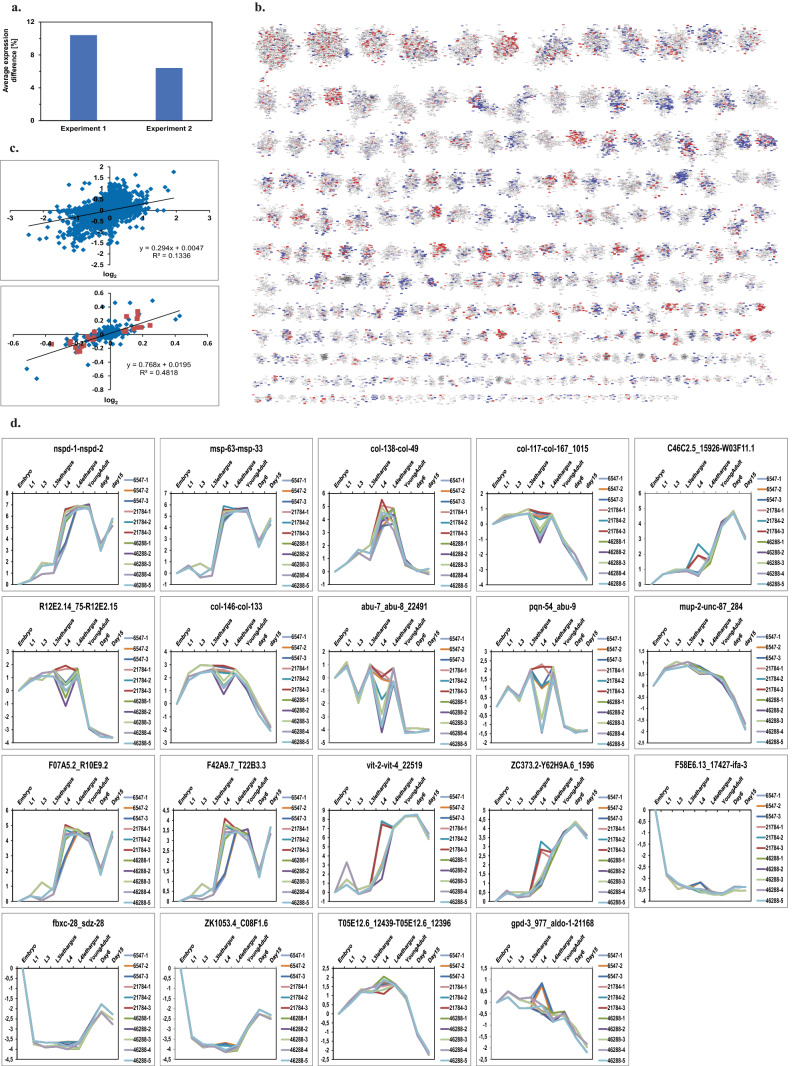
*unc-45* RNAi leads to delayed conclusion of sperm and vulva development. (**a**) Average gene expression difference of the experiments, determined with TAC, compared to the control RNAi. (**b**) Clique map for experiment 1 of *unc-45* RNAi versus young adult of control RNAi. A more detailed description of the most strongly affected cliques can be found in Table 3 and corresponds to the positions in the clique map. (**c**) Comparison between experiment 1 and 2 on a gene-by-gene basis (upper panel) and comparison between experiment 1 and 2 on a clique-by-clique basis (lower panel). The clique map of experiment 2 is shown as Supplemental Fig. 2b, d) Clique trends during development of *C. elegans*. Described interaction between genes involved in sperm development is shown as Supplemental Fig. 2c. Cliques colored in red are induced upon RNAi while cliques in blue are repressed. The linear regression function was generated with Microsoft Excel without weighting, a square value of 1 would indicate a perfect correlation between the cliques.

We then investigated the RNAi treatment against the HSP-90 cofactor *unc-45* with the same approach. *unc-45* RNAi-treatment leads to developmental disruptions and incomplete fertility at a more adult stage. To see, whether differences in the cliques can be observed we performed two independent RNAi-experiments with subsequent transcriptome analysis on DNA microarrays. Analysis with TAC showed a weaker response compared to the *hsp-90* RNAi in both experiments (Fig. 2a, Supplemental Fig. 2a). This also was evident in the analysis of the 307 expression cliques, where the color scheme had to be adjusted to visualize the concerted reactions (Fig. 2b and Supplemental Fig. 2b). Gene–gene comparisons showed a coefficient of determination of 0.15 between the experiments. When cliques were compared a coefficient of determination of 0.48 was obtained (Fig. 2c), confirming that also very weak responses can yield higher levels of repeatability by comparing matched groups of genes and not individual genes.

**Table 3:** Most strongly affected cliques by *unc-45* RNAi and their characteristics.

Cluster name	Clique position	Best GO-Term	Best PEA-Term	Best TEA-Term	Mean STD	Log10(p) Exp 1	Log10(p) Exp 2
nspd-1-nspd-2	R12 C1	Structural constituent of cuticle GO:0042302	Dumpy WBPhenotype:0000583	WBPaper00037950_coelomocytes_embryo_enriched_WBbt_0005751_570	0.38 ± 0.12	4.41	1.84
msp-36-msp-55	R10 C19	Lipid storage GO:0019915	Linker cell migration variant WBPhenotype:0001511	WBPaper00040420_FLP_enriched_WBbt_0006828_288	0.35 ± 0.08	1.15	2.54
msp-63-msp-33	R9 C14	Lipid storage GO:0019915	Linker cell migration variant WBPhenotype:0001511	WBPaper00040420_FLP_enriched_WBbt_0006828_288	0.31 ± 0.09	7.92	9.08
hsp-16.2-F44E5.4_19238	R12 C4	Response to heat GO:0009408	Cadmium response variant WBPhenotype:0001653	WBPaper00037950_coelomocytes_larva_enriched_WBbt_0005751_229	0.29 ± 0.17	16.49	1.60
abu-7-abu-8_22491	R8 C26	Response to unfolded protein GO:0006986	Dauer constitutive WBPhenotype:0000012	WBPaper00024505_pharyngeal_enriched_WBbt_0003681_329	0.25 ± 0.08	63.31	5.15
col-138-col-49	R9 C23	Structural constituent of cuticle GO:0042302	Blistered WBPhenotype:0000025	WBPaper00037950_coelomocytes_larva_enriched_WBbt_0005751_229	0.24 ± 0.22	34.90	0.93
col-117-col-167_1015	R9 C25	Structural constituent of cuticle GO:0042302	Dumpy WBPhenotype:0000583	WBPaper00037950_germline-precursors_embryo_enriched_WBbt_0006849_974	0.24 ± 0.06	29.40	5.29
pqn-54-abu-9	R6 C11	Response to unfolded protein GO:0006986	Shortened life span WBPhenotype:0001171	WBPaper00024505_pharyngeal_enriched_WBbt_0003681_329	0.2 ± 0.03	43.57	13.10
R12E2.14_75-R12E2.15	R6 C9	Structural constituent of cuticle GO:0042302	Dumpy WBPhenotype:0000583	WBPaper00037950_germline-precursors_embryo_enriched_WBbt_0006849_974	0.18 ± 0.08	47.12	8.99
lys-3-tsp-1	R11 C40	Carbohydrate metabolic process GO:0005975	Male nervous system development variant WBPhenotype:0001008	WBPaper00040420_ALM_PLM_enriched_WBbt_0005406_198	0.18 ± 0.22	27.93	1.06
C10C5.3-C10C5.5	R12 C40	Oxoacid metabolic process GO:0043436	Dauer constitutive WBPhenotype:0000012	WBPaper00037950_pharyngeal-muscle_embryo_enriched_WBbt_0005451_598	0.17 ± 0.05	4.43	2.28
F07A5.2-R10E9.2	R5 C7	Sodium ion transport GO:0006814	Nicotine response variant WBPhenotype:0001573	WBPaper00045521_Spermatogenic_WBbt_0005784_2743	0.15 ± 0.04	6.82	34.38
K11C4.1_20445-rnh-1.1	R8 C22	Regulation of cell shape GO:0008360	Fat content increased WBPhenotype:0001184	WBPaper00045521_Spermatogenic_WBbt_0005784_2743	0.14 ± 0.03	6.02	8.77
ssq-2_16507-ssq-3_1032	R12 C27	Response to hormone GO:0009725	Movement variant WBPhenotype:0001206	WBPaper00026980_intestine_enriched_WBbt_0005772_1970	0.13 ± 0	1.96	2.06
col-146-col-133	R9 C28	Structural constituent of cuticle GO:0042302	Dumpy WBPhenotype:0000583	WBPaper00037950_coelomocytes_larva_enriched_WBbt_0005751_229	0.12 ± 0.02	11.00	2.77
F42A9.7-T22B3.3	R3 C11	Regulation of cell shape GO:0008360	Dauer metabolism variant WBPhenotype:0001547	WBPaper00045521_Spermatogenic_WBbt_0005784_2743	0.11 ± 0.03	7.97	29.43
bus-8_3160-K04H4.2_2324	R9 C2	Amino sugar metabolic process GO:0006040	Molt variant WBPhenotype:0002041	WBPaper00024505_pharyngeal_enriched_WBbt_0003681_329	0.11 ± 0.08	8.94	0.92
R12A1.3-M195.2	R6 C19	Amino sugar metabolic process GO:0006040	Dauer constitutive WBPhenotype:0000012	WBPaper00024505_pharyngeal_enriched_WBbt_0003681_329	0.1 ± 0.04	6.65	3.63
T28A11.5-T06C12.14	R12 C14	Extracellular region GO:0005576	Dumpy WBPhenotype:0000583	WBPaper00037950_excretory-cell_larva_enriched_WBbt_0005812_528	0.1 ± 0.05	0.71	2.01
agmo-1_5527-F53B1.4	R10 C14	Pyridoxal phosphate binding GO:0030170	Molt variant WBPhenotype:0002041	WBPaper00037950_hypodermis_embryo_enriched_WBbt_0005733_734	0.1 ± 0.11	7.44	0.70
171971_x_at-D1054.11_184	R11 C37	Cell GO:0005623	Egg laying defective WBPhenotype:0000006	WBPaper00037950_hypodermis_larva_enriched_WBbt_0005733_1250	− 0.55 ± 0.09	3.70	0.66
gpd-3_977-aldo-1_21168	R9 C26	Glycosyl compound metabolic process GO:1901657	Fat content reduced WBPhenotype:0001183	WBPaper00031003_24hr_muscle_enriched_WBbt_0003675_918	− 0.51 ± 0.01	9.02	4.52
T05E12.6_12439-T05E12.6_12396	R10 C3	Lipid catabolic process GO:0016042	Transgene expression increased WBPhenotype:0001236	WBPaper00037950_pharyngeal-muscle_embryo_enriched_WBbt_0005451_598	− 0.29 ± 0.15	60.37	3.63
ZK1053.4-C08F1.6	R4 C16	Embryo development GO:0009790	Embryonic development variant WBPhenotype:0000749	WBPaper00037950_hypodermis_embryo_enriched_WBbt_0005733_734	− 0.26 ± 0.12	26.51	73.26
C46C2.5_15926-W03F11.1	R9 C22	Carbohydrate binding GO:0030246	Apoptosis increased WBPhenotype:0000183	WBPaper00037950_GABAergic-motor-neurons_embryo_enriched_WBbt_0005190_361	− 0.24 ± 0.02	8.57	10.00
ZC373.2-Y62H9A.6_1596	R3 C18	Flavonoid metabolic process GO:0009812	Cell membrane organization biogenesis variant WBPhenotype:0001982	WBPaper00037950_dopaminergic-neurons_larva_enriched_WBbt_0006746_1230	− 0.22 ± 0.03	46.68	19.54
nspc-1_614-nspc-10_22525	R10 C12	Extracellular region GO:0005576	Spermatogenesis variant WBPhenotype:0000670	WBPaper00037950_coelomocytes_larva_enriched_WBbt_0005751_229	− 0.21 ± 0.06	5.62	5.79
C46C2.5_15925-F17E9.2	R10 C8	Hydrolase activity—acting on glycosyl bonds GO:0016798	Embryonic development variant WBPhenotype:0000749	WBPaper00037950_coelomocytes_larva_enriched_WBbt_0005751_229	− 0.2 ± 0.01	6.65	6.25
C03B1.14-F46C3.2	R8 C21	Membrane GO:0016020	Chemical hypersensitive WBPhenotype:0001918	WBPaper00037950_intestine_larva_enriched_WBbt_0005772_946	− 0.19 ± 0	9.53	10.38
fbxc-28-sdz-28	R7 C8	Modification-dependent macromolecule catabolic process GO:0043632	L1 larval development variant WBPhenotype:0000751	WBPaper00037950_hypodermis_embryo_enriched_WBbt_0005733_734	− 0.18 ± 0.08	10.65	27.90
fbxb-13-fbxb-24	R8 C18	Protein oligomerization GO:0051259	Cholinergic agonist resistant WBPhenotype:0001578	WBPaper00024505_pharyngeal_enriched_WBbt_0003681_329	− 0.17 ± 0.1	1.20	13.08
dsh-1_3575-C40A11.4	R5 C17	Protein oligomerization GO:0051259	Ectopic expression transgene WBPhenotype:0001276	WBPaper00037950_hypodermis_embryo_enriched_WBbt_0005733_734	− 0.14 ± 0.07	2.78	9.72
vem-1-ugt-58	R12 C35	Oxoacid metabolic process GO:0043436	Epithelial cell physiology variant WBPhenotype:0000986	WBPaper00037950_BAG-neuron_embryo_enriched_WBbt_0006825_454	− 0.14 ± 0.01	2.03	0.89
sdz-10-fbxb-62	R3 C17	Glycosylation GO:0070085	L1 larval development variant WBPhenotype:0000751	WBPaper00037950_hypodermis_embryo_enriched_WBbt_0005733_734	− 0.14 ± 0.09	2.82	41.16
Y41D4B.17-K10D11.6	R12 C32	Immune system process GO:0002376	Exploded through vulva WBPhenotype:0000038	WBPaper00037950_intestine_larva_enriched_WBbt_0005772_946	− 0.12 ± 0.03	0.84	0.77
fbxb-31-fbxb-119	R11 C28	Embryo development GO:0009790	Transgene expression reduced WBPhenotype:0001278	WBPaper00040420_FLP_enriched_WBbt_0006828_288	− 0.12 ± 0.09	0.64	4.30
R03E1.2_7363-ucr-2.1	R10 C30	Mitochondrion GO:0005739	mRNA levels increased WBPhenotype:0000136	WBPaper00037950_excretory-cell_larva_enriched_WBbt_0005812_528	− 0.12 ± 0.03	0.98	2.14
C35C5.8_15869-best-1	R10 C21	Transmembrane transport GO:0055085	Fat content increased WBPhenotype:0001184	WBPaper00037950_excretory-cell_larva_enriched_WBbt_0005812_528	− 0.12 ± 0.03	4.11	1.04
pek-1_22220-pek-1_33	R11 C12	Aging GO:0007568	Sluggish WBPhenotype:0000646	WBPaper00037950_GABAergic-motor-neurons_embryo_enriched_WBbt_0005190_361	− 0.11 ± 0	1.85	1.69
iff-2_18754-rpl-25.1	R12 C37	Amide biosynthetic process GO:0043604	Hermaphrodite fertility reduced WBPhenotype:0001259	WBPaper00037950_PVD-OLL-neurons_larva_enriched_WBbt_0006831_878	− 0.11 ± 0.03	0.65	1.39

Clique positions (letter = row, number = position from left to right) correspond to the clique map shown in Fig. 2b.

Like with *hsp-90* RNAi, specific cliques were found in all experiments to be significantly altered in their expression behavior. Upregulated are a few smaller cliques, like col-117-col-167_1015, msp-63-msp-33 and abu-7-abu-8_22491. These represent decisions to produce cuticle collagens, linker cell movement and induction of a response to heat. This also is reflected by the induction of hsp-16.2-F44E5.4_19238, a small clique containing the heat-shock proteins. This induction of the heat-shock response has been observed for *unc-45* RNAi before^7^. Downregulated cliques represent the large cliques ZK1053.4-C08F1.6 (embryo development), ZC373.2-Y62H9A.6_1596 (cell membrane biogenesis) and sdz-10-fbxb-62 (L1 larval development). The weak differences, while being statistically significant for the described cliques, correlate with the mostly adult state of the nematode after *unc-45* RNAi treatment and the observation that most developmental steps were performed, but the correct embryo development and the development of the vulva structure were affected^7^.

A hint towards the lacking germ line development may be derived from the misregulation of the cliques msp-36_msp-63 (linker-cell migration variant), which contains several genes related to sperm development and the downregulation of nspc-1_614-nspc-10_22525 (spermatogenesis variant). Thus, while vulva development and sperm development are stalled, certain features of the regulatory pathways are not deviating from the control nematodes and only later stages of the programmatic decision process show deviations that could explain the mismanaged development in the absence of *unc-45*.

### Expression in developmental stages is altered in similarity to the RNAi-induced arrest

Having observed cliques with altered expression, we aimed at understanding, whether these expression changes are specific for one developmental transition occurring at the time point of arrest. We thus generated a time series of development ranging from embryo to late adult and compared the expression of all 307 cliques and in particular of those found relevant for *unc-45* RNAi.

Striking differences were observed, when comparing the stages of each series (Supplemental Fig. 3), while differences between experiments of the same stage were small (Supplemental Figs. 4 and 5). Interestingly, also in these comparisons most of the isolated expression cliques showed coordinated expression differences, and also strong responses could be observed for the later developmental stages (Supplemental Fig. 6). In total more then 80% of the cliques show a statistically significant expression change during the development from embryo to 16 day adult and this also relates to most cliques found affected after *unc-45* RNAi (Fig. 2d, 2 cliques and their development). While only few cliques were affected upon *unc-45* RNAi treatment, *hsp-90* RNAi is expected to yield a much stronger response.

Indeed a drop is observed in the expression of most upregulated cliques between L4 and day6 adult. In these cases the developmental delay may be the reason of the observed higher expression. A opposite pattern is observed for the downregulated gene cliques, with the exception of two cliques, which are not appropriately regulated: T05E12.6_12439-T05E12.6_12396 and gpd-3_977-aldo-1_21168, both of which appear to regulate metabolism.

### Expression in developmental stages is altered in similarity to the *hsp-90* RNAi-induced arrest

**Figure 3:**
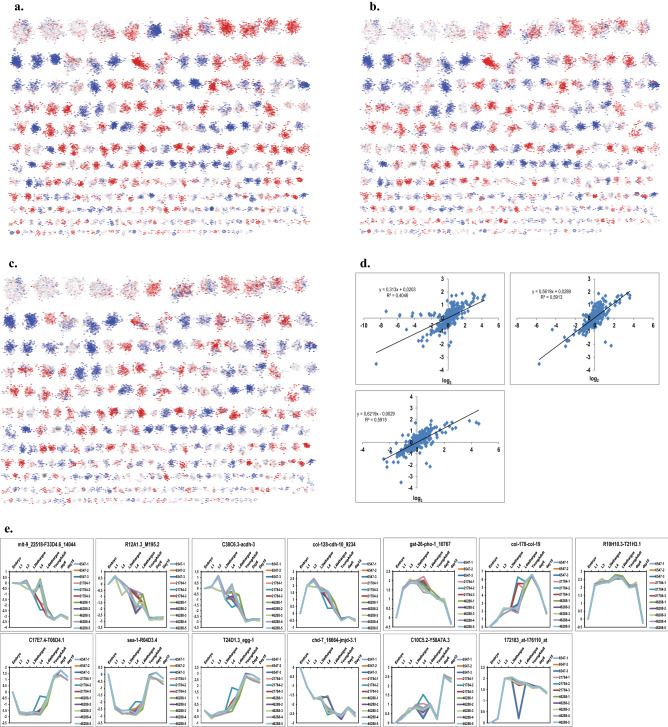
Expression in developmental stages is altered in similarity to the RNAi-induced arrest. (**a**) L3 comparison to young adult. (**b**) L4 comparison to young adult and (**c**) L4 lethargus to young adult. Other stepwise comparisons, like that of embryo to L1 or that of day6 nematode to day 16 adult nemtode are shown in the Supplemental Figures section as Supplemental Fig. 3, 4, 5 and 6. (**d**) Clique responses throughout development shown for selected cliques with significant change pattern. Cliques colored in red are induced compared to the earlier developmental stage, while cliques colored in blue are repressed. The linear regression function was generated with Microsoft Excel without weighting, a square value of 1 would indicate a perfect correlation between the cliques.

We next tested, whether also for the *hsp-90* RNAi-treated nematodes developmental stages can be defined. The complexity of the differential expression between *hsp-90* RNAi arrested nematodes and young adults allow to compare the obtained expression patterns with know patterns from larval development. We thus were interested to see, whether the full extent of the transcriptional changes can be explained by the observed developmental delay. Therefore we utilized publicly available microarray experiments on nematode development to help identify transcriptional units in the clique map that report on comparable steps during development. We employed microarray data from three experimental series (Table 4) and initially compared developmental transitions, showing similarity to the differences we observe in the RNAi-treated nematodes. These comparisons were L3/young adult, L4/young adult and L4let/young adult (Fig. 3a–c) as investigated in GSE46288/GSE46289^28^. Clearly similarities can be observed between the *hsp-90* RNAi treated nematodes and the L4 larvae, when each of them is compared to the young adult control. In fact, most of the cliques correlate in color and correlation analysis shows a coefficient of determination with these data of 0.4046, 0.5913 and 0.5915 (Fig. 3d). Based on these values, *hsp-90* RNAi-arrested nematodes best correspond to a L4-larval like state. Only few clear differences can be observed compared to L4 or L4-lethargus, while several cliques deviate from L3-like state. Judged from the few differences to L4 state, it might be that the chronological timing of the events during development is misaligned in *hsp-90* RNAi-arrested nematodes.

**Table 4:** Experiments used for the analysis of the developmental time line of *C. elegans*, obtained from the GEO expression data repository.

Series	Sample	Description	Replicate
GSE6547	GSM146422	N2 worms at L1 stage	1
	GSM146423	N2 worms at L1 stage	2
	GSM147330	N2 worms at L1 stage	3
	GSM147334	N2 worms at L4 stage	1
	GSM147335	N2 worms at L4 stage	2
	GSM147336	N2 worms at L4 stage	3
	GSM147340	N2 worms at embryonic stage	1
	GSM147341	N2 worms at embryonic stage	2
	GSM147342	N2 worms at embryonic stage	3
GSE21784	GSM542652	L4 larvae	1
	GSM542653	L4 larvae	2
	GSM542654	L4 larvae	3
	GSM542655	Day 6 adult	1
	GSM542656	Day 6 adult	2
	GSM542657	Day 6 adult	3
	GSM542658	Day 15 adult	1
	GSM542659	Day 15 adult	2
	GSM542660	Day 15 adult	3
GSE46288	GSM1128166	L3	1
	GSM1128167	L3	2
	GSM1128168	L3	3
	GSM1128169	L3-lethargus	1
	GSM1128170	L3-lethargus	2
	GSM1128171	L3-lethargus	3
GSE46289	GSM1128172	L4	1
	GSM1128173	L4	2
	GSM1128174	L4	3
	GSM1128175	L4	4
	GSM1128176	L4	5
	GSM1128177	L4-lethargus	1
	GSM1128178	L4-lethargus	2
	GSM1128179	L4-lethargus	3
	GSM1128180	L4-lethargus	4
	GSM1128181	L4-lethargus	5
	GSM1128182	Adult	1
	GSM1128183	Adult	2
	GSM1128184	Adult	3
	GSM1128185	Adult	4
	GSM1128186	Adult	5

We further investigated, whether the expression behavior matches the known expression behavior during development and aging. To this end the cliques found relevant for *hsp-90* RNAi were investigated throughout development. While the strongly responsive upregulated cliques col-138_col-49, abu-7_abu-8, R12E2.14_75-R12E2.15, col-117_col-167, pqn-54_abu-9, col-146_col-133 already were found in context with the *unc-45* RNAi-arrested state (see Fig. 2d), further cliques are identificed as significantly upregulated in *hsp-90* RNAi treated nematodes (Fig. 3e). As for *unc-45* RNAi before the downregulated cliques vit-2_vit-4, C46C2.5_W03F11, ZC373.2-Y62H9A.6_1596, ZK1053.4-C08F1.6 were identified amid fbxc-28_sdz-28 (see Fig. 2d), plus additional ones not found significant before (Fig. 3e). Interestingly, in few cases, the directionality of the RNAi-induced response does not correlate with the expected behaviour during the arrested L4 state. This is evident for the cliques C17H1.6-C17H1.13, C32F10.4-D1086.2 and C10C5.2-Y58A7A.3.

### *daf-16* target genes deviate from the developmental program in *hsp-90* RNAi

**Figure 4:**
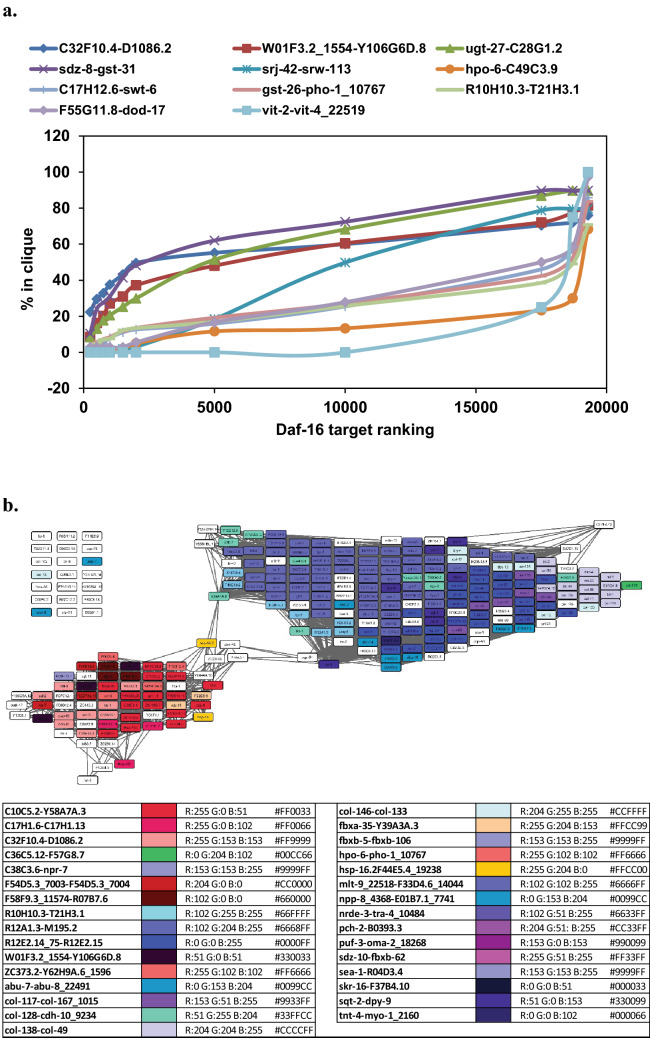
Correlation between *daf-16* target genes and identified cliques for those genes most clearly enriched in *daf-16* targets. (**a**) Enrichted target cliques of DAF-16. The plot shows how many genes per clique are derived from the indicated range of the DAF-16 ranking. Cliques with high percentage values on the left side reflect cliques that are considered to be DAF-16 activatable while cliques with a low percentage up to the bottom to the DAF-16 ranking are considered repressed. (**b**) Most strongly enriched cliques in the Top750 and Bottom750 of the ranked *daf-16* target list.

We finally aimed at understanding the few cliques that deviate from the developmental progression and the explanation based on developmental delays. To this end we used the information gained previously that a fraction of the misregulated genes are *daf-16* targets^11^. We tested, which of the cliques from the clique map contain *daf-16* targets and then tested, whether those are regulated in coordance with developmental progress. Indeed, targets upregulated and suppressed by DAF-16 are enriched in several cliques (the 15 most prominent shown in Table 5, more information in Fig. 4a and b). Comparing the clusters identified in Eckl et al*.* (2017), with the current cliques we also observe a clear enrichment among several of the 307 cliques (Table 5). As spectulated in Eckl et al*.*, among the cluster “Up1” there are many genes, which are regulated by DAF-16, while cluster “Up2” does not enrich *daf*-16 targets (Table 5). Mapping all cliques onto the network developed in Eckl et al. the enrichment of these cliques in certain parts of the network becomes evident. For the downregulated genes, also DAF-16 enriching cliques are among those containing these genes^11^. Therefore, especially among the upregulated genes, cliques are present, which contain an elevated level of *daf-16* target genes.

**Table 5:** HSP-90-responsive cliques with highest enrichment of *daf-16* supported (red) and suppressed (blue) genes.

Up1	Up2	Down1	Down2	Down3	Down4
C10C5.2-Y58A7A.3	col-138-col-49	C46C2.5_15926-W03F11.1	C17E7.4-T06D4.1	vit-2-vit-4_22519	C17H12.6-swt-6
K08D10.9-F46A8.1	R12E2.14_75-R12E2.15	ZC373.2-Y62H9A.6_1596	fbxc-28-sdz-28	C10A4.6-C01A2.6	F55G11.8-dod-17
hsp-16.2-F44E5.4_19238	mlt-9_22518-F33D4.6_14044	C10C5.3-C10C5.5	sea-1-R04D3.4	fbxc-28-sdz-28	R10H10.3-T21H3.1
lys-3-tsp-1	col-117-col-167_1015	171971_x_at-D1054.11_184	ZK1053.4-C08F1.6	C17E7.4-T06D4.1	
hpo-6-C49C3.9	col-146-col-133	C46C2.5_15925-F17E9.2	F11A3.2-F47G3.3	R07E4.5-skp-1_1608	
C32F10.4-D1086.2	bus-8_3160-K04H4.2_2324	C03B1.14-F46C3.2	K09D9.12-T10C6.10	R10H10.3-T21H3.1	
F13E6.1_2090-C35C5.3_1843	sqt-2-dpy-9	col-178-col-19	K05C4.4-fbxc-46	sre-33-ZK1025.1_8337	
Y6G8.2_5650-F17B5.1	abu-7-abu-8_22491	T24D1.3-egg-1	fbxb-31-fbxb-119		
fbxa-35-Y39A3A.3	agmo-1_5527-F53B1.4		Y116A8C.19-F38C2.7		
C17H1.6-C17H1.13	dos-2-grd-2		npp-8_4368-E01B7.1_7741		
ccg-1_12351-ZC410.5	R12A1.3-M195.2		sdz-10-fbxb-62		
C17H12.6-swt-6	pqn-54-abu-9		fbxb-13-fbxb-24		
F58F9.3_11574-R07B7.6	col-128-cdh-10_9234		skr-16-F37B4.10		
T12G3.1_17-T12G3.1_18846	C38C6.3-acdh-6		dsh-1_3575-C40A11.4		
W01F3.2_1554-Y106G6D.8	C36C5.12-F57G8.7		dos-2-grd-2		

Sorted according to the nomenclature estabilshed in Eckl et al., 2017, which grouped the *hsp-90* responsive genes in two major groups for upregulation and four groups for downregulation based on co-expression network analysis. Cliques enriched within these groups are listed under the group names (Up1, Up2, Down1, Down2, Down3 and Down4). The groups are labeled in bold, if enrichment reaches the significance level of 1e-5 and they are colored, if the same group is found within the top *daf-16* regulated groups. Coloring is red, if it is among upregulated DAF-16 targets and blue if, it is among down regulated DAF-16 targets.

Interestingly, these cliques are upregulated despite their developmental program, which aims for downregulation. Thus, the presence of these cliques suggests a simultaneous modification to the dauer-program outside the developmental program after *hsp-90* RNAi induced growth arrest.

## Discussion

In this study, we analysed microarray data from *C. elegans* based on preformed coregulated expression cliques. This approach has been applied successfully in the yeast model organism by us^17^, but the applicability of this method to multicellular organisms has not been clear. We thus used the algorithms developed for yeast to also generate high quality coexpression cliques from nematode expression data and then validated them by GO-term enrichment, phenotype enrichment and tissue enrichment and by selective clique responses in individual microarray experiments. Based on our data from the developmental process of *C. elegans*, we believe that this analysis method could have broader use in the analysis of gene expression data from nematodes. This is evident from the correlated responses of cliques during nematode developmental transitions.

Recently also a different approach was reported to utilize genome-wide co-expression cliques for *C. elegans*^29^. In contrast to our appraoch, in this study an individual gene could be assigned to multiple cliques (on average 3) and also negative correlation was included. This makes the construction of a static clique map as used by us more difficult, but may include details missed by our approach. Both approaches will have their advantages. In the method described by us, we focus on the strongest connection and blank out those that might be secondary based on numbers, but still achieve very high levels of correlation with GO-terms, phenotypes and tissue specificity for most of the cliques.

One way to use the cliques could be by employing the popular GSEA platform^30^, where our cliques can be either used as a single input file covering the whole genome or as part of the global collection of gene sets. Another way to use the cliques can also be via the clusterEX.de webserver that we have set up and will further develop for the purpose of gene expression analysis based on known co-expression relationships. It therefore will be interesting to see, how further useful applications will be developed based on these predefined gene sets.

### Integrating *unc-45* into the developmental time line exposes distinct cliques for develeopmental stop

We first analysed *unc-45* depleted nematodes. In these nematodes, the depletion of *unc-45* leads to developmental arrest and paralysis in almost adult animals. Here the comparison with the young adult nematode shows that certain cliques are misregulated and some of those cliques also represent developmental marker cliques as suggested by our evaluation procedure. These marker genes help to map the developmental status of the *unc-45* depleted organisms. Clearly *unc-45* depleted nematodes are close to N2 nematodes in this approach, but defined changes in certain genes help to map the events that did not unfold during development.

To evaluate the disruption of vulva development, we individually tested the genes transcriptionally regulated during this process and their specific regulation (Table 3): *eff-1* (log2(dExp) = 0.185), *egl-18* (− 0.035), *egl-17* (0.000), *lin-3* (− 0.015), *lin-31* (0.00), *lin-39* (0.00), *egl-30* (0.02), *lag-2* (− 0.09), *apx-1* (0.055), *dsl-1* (− 0.085 as part of fbxc-28-sdz-28) and *elt-6* (-0.065), all of which are getting induced during vulva development^31^. In a critical step during vulva development the VPCs express LIN-39, which together with its cofactors CEH-20 and UNC-62, activates the expression of *ref-2*, which inhibits the expression of the fusogen EFF-1^32^). In UNC-45 depleted nematodes, *ref-2* is not yet upregulated compared to mock treated nematodes (− 0.675 and resides in clique cfz-2_18944-cfz-2_2268, which is downregulated twice significantly, but not very strongly) and also *ref-1* is lower expressed in *unc-45* RNAi-treated nematodes (− 0.46, ZK1053.4-C08F1.6), even though *lin-39* is expressed as in the control and *eff-1* is higher expressed (0.185, tnt-4-myo-1_2160), as expected for vulva development^33^. Thus, based on these expression patterns the induction to generate the vulva is not transmitted properly by the anchor cell from the developing gonad. Also *lin-12* (− 0.31, sol-1-jnk-1_18695), *cwn-1* (− 0.26, chd-7_16664-jmjd-3.1) and *vang-1* (− 0.175, nrde-3-tra-4_10484) are downregulated, further implying that central decisions to induce the vulva have not been made yet.

Regarding the germline, *asb-2* is reduced (− 0.21, tars-1-AFFX-r2-3026-5_at) and the nspd-proteins are still upregulated together with msp-proteins (Fig. 2d^34^), implying that sperm development is not completed yet, while the expression of the upstream regulators *spn-4* and *neg-1*^35^ is at the same level as in the normally developed adult. Also the regulators of msp-expression *set-17* and *csr-1* are expressed at the level of the control nematodes^36^, implying that sperm-development is almost finished^37,38^.

### Integrating *hsp-90* into the time line data exposes defined clusters for developmental stop

We used this clique map to also analyse the depletion of *hsp-90*. While depletion of *hsp-90* leads to developmental arrest and reduced motility in late larval stages, it also leads to defined transcriptional changes. To analyse the causes, we performed microarray experiments under wildtype conditions and under conditions, where the chaperone is depleted. Based on the clique analysis, it is obvious that certain developmental milestones are not reached yet in the HSP-90 depleted animals. Based on this analysis these nemtodes arrest in a late larval stage with additional misregulation of DAF-16 target genes.

Previously it had been observed that the Top300 genes from the *hsp-90* RNAi analysis showed partial overlap with *daf-16* regulated genes. We thus employed the gene-list from this previous study to identify the cliques, which now represent these genes. Indeed the correlation is fairly clear, with the cliques C17H1.6-C17H1.13, C32F10.4-D1086.2 and C10C5.2-Y58A7A.3 being mostly overlapping with the previous cluster1_up and the cliques col-138-col-49, R12E2.14_75-R12E2.15, mlt-9_22518-F33D4.6_14044 being mostly overlapping with the cluster2_up. Utilizing the ranked list of *daf-16* target genes, we also determined which cliques most strongly are enriched in the Top750 and Bottom750 of this ranked list. These cliques are found mostly in cluster1_up confirming that the identification of this correlation also is visible from the clique map. Interestingly these cliques represent those that are differently regulated compared to the L4 larval stage. Thus the HSP-90 depletion leads to higher exression levles in a *daf-16* regulated cluster (cluster1_up) and a *daf-16* independent cluster (cluster2_up). With the *daf-16* independent cluster containing mostly cliques related to larval development, apparently the depletion of HSP-90 induces both of these processes. Whether they are connected via secondary effects is unclear to date, especially as the developmental timing of DAF-16 activity is a well described phenomenon.

Thus, based on several clearly regulated marker cliques, *hsp-90* arrested nematodes, like *unc-45* arrested nematodes, can be positioned in respect to a developmental time axis.

## Supplementary Information

Supplementary Information 1.

Supplementary Information 2.

Supplementary Information 3.

Supplementary Information 4.

Supplementary Information 5.

Supplementary Information 6.

Supplementary Information 7.

Supplementary Information 8.

Supplementary Table.

## Data Availability

All data will be made fully available without restriction at http://www.richterlab.de/DataSets and on the GEO repository. Tables containing GO terms, PEA and TEA enrichment results for all cliques can be obtained from the authors.
